# Supplementary material for: Evaluation of Antifouling Potential and Ecotoxicity of Secondary Metabolites Derived from Red Algae of the Genus Laurencia
Source: Mar Drugs. 2019 Nov 16;17(11):646. doi: 10.3390/md17110646 (PMC6891695; doi:10.3390/md17110646)
Supplement: Supplementary file 1 [file marinedrugs-17-00646-s001.pdf]

Supplementary Materials for

Evaluation of Antifouling Potential and Ecotoxicity of Secondary Metabolites Derived from Red Algae of the Genus *Laurencia*

Maria Protopapa [1], Manto Kotsiri [1], Sofoklis Mouratidis [1], Vassilios Roussis [2], Efstathia Ioannou [2,*] and Skarlatos G. Dedos [1,*]

[1] Department of Biology, National and Kapodistrian University of Athens, Panepistimiopolis Zografou, Athens 15784, Greece; mariaprot@hotmail.com (M.P.); madokot@yahoo.gr (M.K.); aesofos@gmail.com (S.M.)

[2] Section of Pharmacognosy and Chemistry of Natural Products, Department of Pharmacy, National and Kapodistrian University of Athens, Panepistimiopolis Zografou, Athens 15771, Greece; roussis@pharm.uoa.gr (V.R.)

* Correspondence: eioannou@pharm.uoa.gr (E.I.); sdedos@biol.uoa.gr (S.G.D.); Tel.: +30-210-727-4913 (E.I.); +30-210-727-4705 (S.G.D.)

Table of Contents

Figure S1. Settlement bioassays of *A. amphitrite* cyprids in the presence of varying concentrations of 25 secondary metabolites isolated from *Laurencia* and *Aplysia* species. S3
Figure S2. Mortality of *A. amphitrite* cyprids in the presence of varying concentrations of 25 secondary metabolites isolated from *Laurencia* and *Aplysia* species. S4
Figure S3. Inhibition of metamorphosis of *A. amphitrite* cyprids in the presence of varying concentrations of 25 secondary metabolites isolated from *Laurencia* and *Aplysia* species. S5
Figure S4. Results of settlement bioassays of *A. amphitrite* cyprids in the presence of varying concentrations of bromosphaerol. S6
Figure S5. Results of *Artemia salina* toxicity assays in the presence of 8 secondary metabolites isolated from *Laurencia* and *Aplysia* species and bromosphaerol. S7
Figure S6. Results of *Chaetoceros gracilis* toxicity assays in the presence of 6 secondary metabolites isolated from *Laurencia* and *Aplysia* species and bromosphaerol. S8
Figure S7. Results of RTL-W1 cell viability assays in the presence of 3 secondary metabolites isolated from *Laurencia* and *Aplysia* species and bromosphaerol. S9
Figure S8. Results of HEK293 cell viability assays in the presence of 3 secondary metabolites isolated from *Laurencia* and *Aplysia* species and bromosphaerol. S10
Figure S9. Results of marine bacteria growth assays in the presence of 3 secondary metabolites isolated from *Laurencia* and *Aplysia* species and bromosphaerol. S11

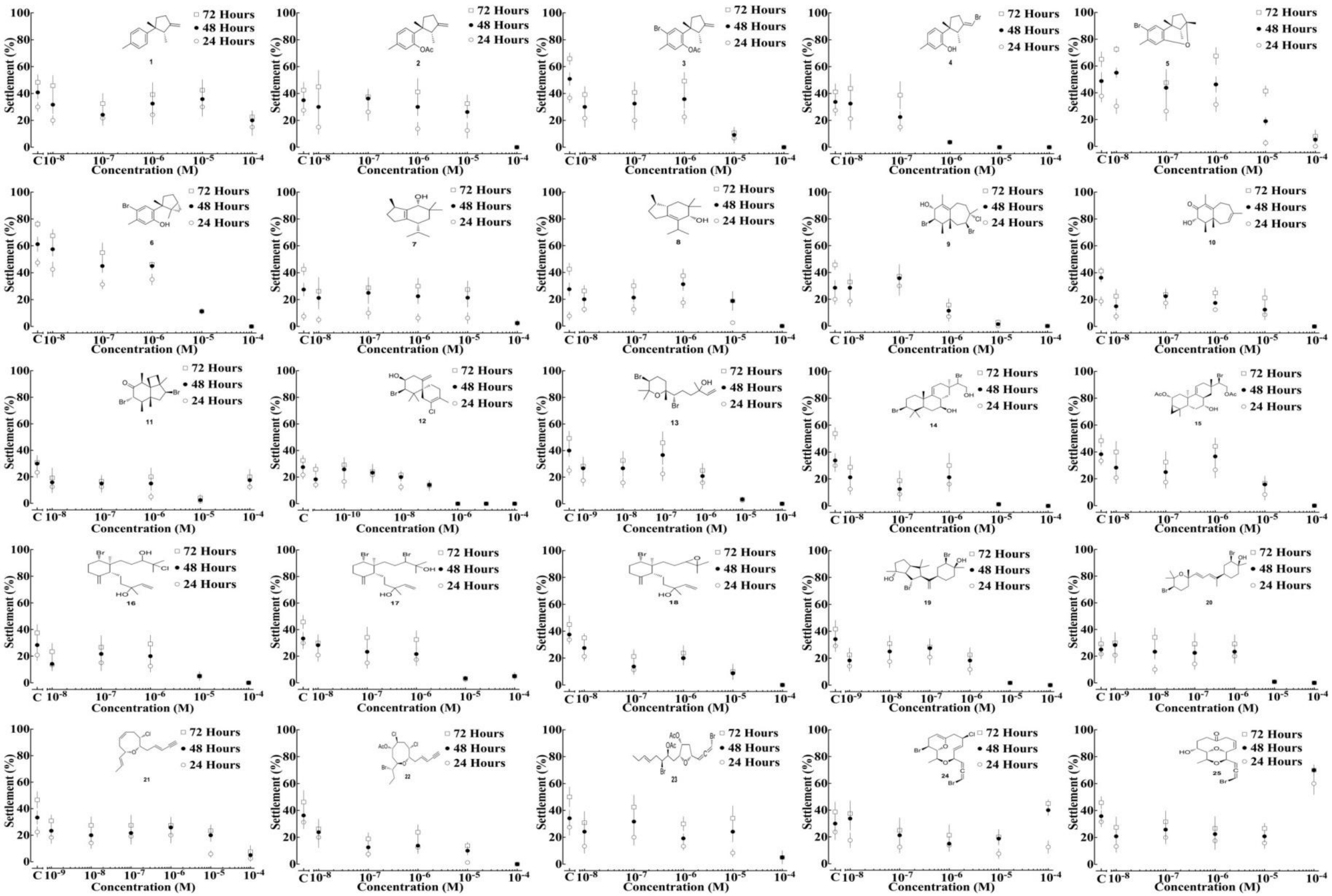

Figure S1. Settlement bioassays of *A. amphitrite* cyprids in the presence of varying concentrations of 25 secondary metabolites isolated from *Laurencia* and *Aplysia* species.

Values are expressed as the percentage of animals that settled from the total number of animals placed in a well of a 24-well tissue culture plate with varying concentrations of the indicated metabolites. The cumulative results from 3-4 independent experiments with 5 replicates each is shown.

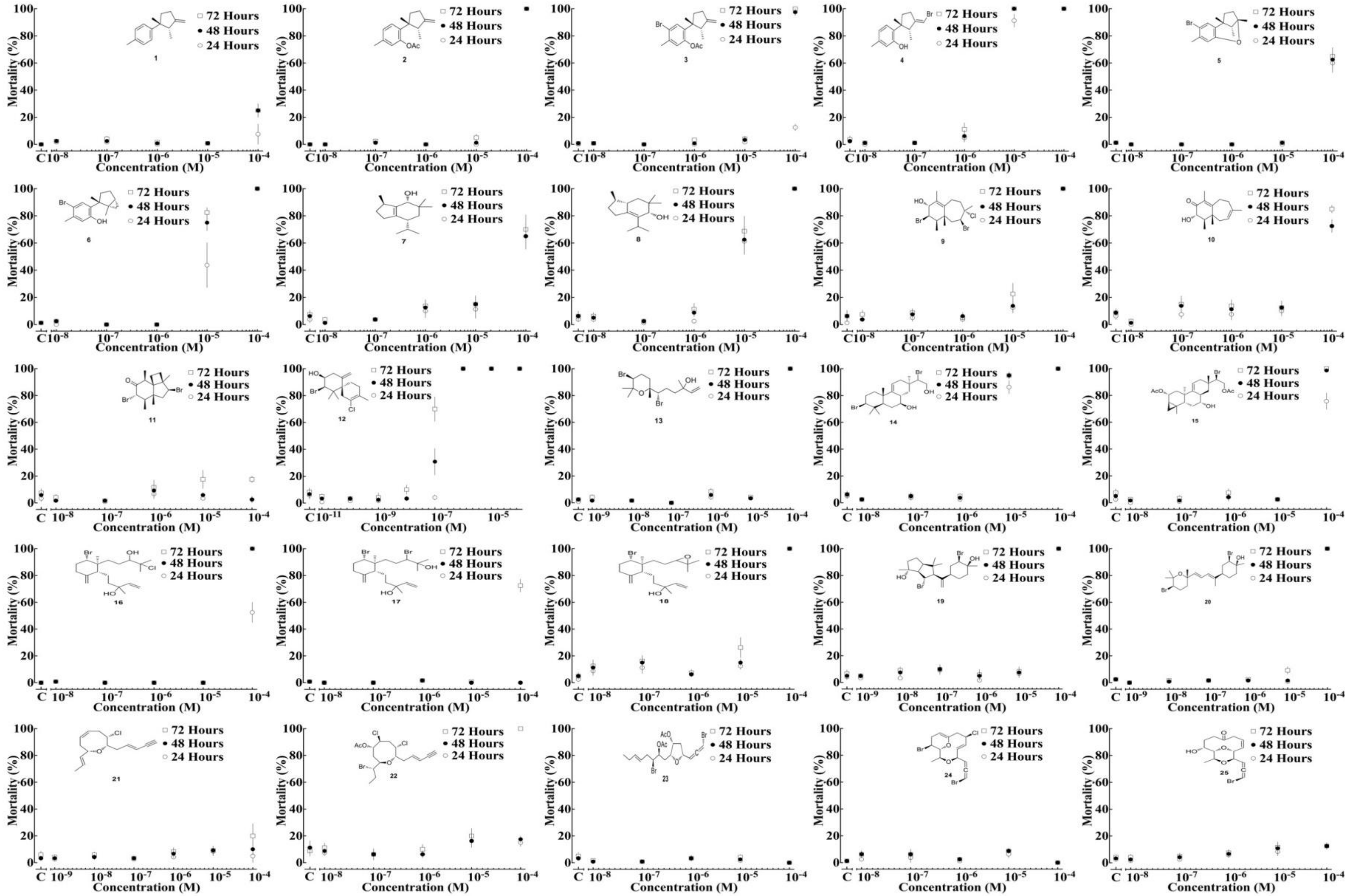

Figure S2. Mortality of *A. amphitrite* cyprids in the presence of varying concentrations of 25 secondary metabolites isolated from *Laurencia* and *Aplysia* species. Values are expressed as the percentage of animals that did not react to light mechanical stimuli from the total number of animals placed in a well of a 24-well tissue culture plate with varying concentrations of the indicated metabolites. The cumulative results from 3-4 independent experiments with 5 replicates each is shown.

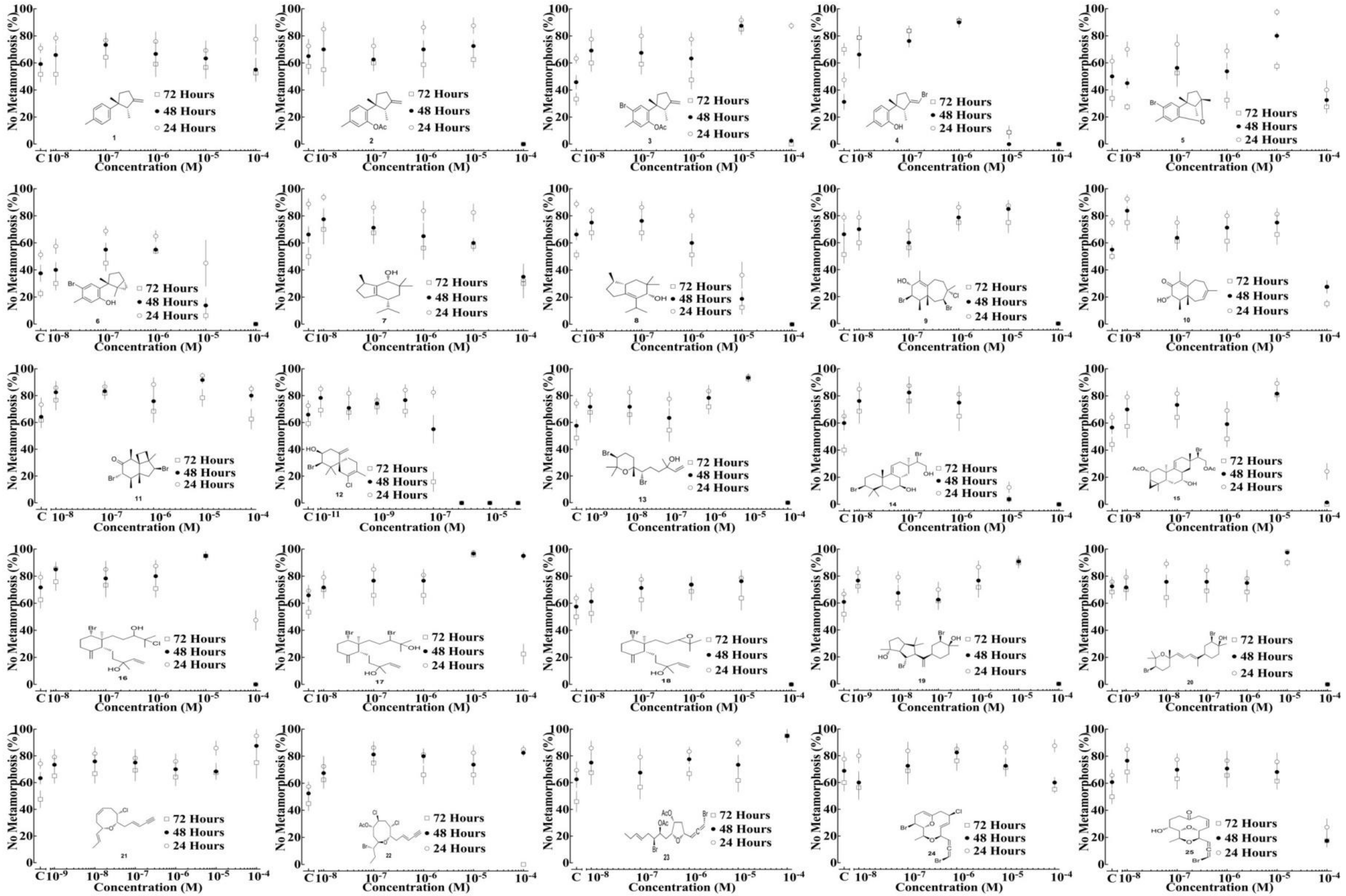

Figure S3. Inhibition of metamorphosis of *A. amphitrite* cyprids in the presence of varying concentrations of 25 secondary metabolites isolated from *Laurencia* and *Aplysia* species.

Values are expressed as the percentage of animals that neither settled, nor were dead from the total number of animals placed in a well of a 24-well tissue culture plate with varying concentrations of the indicated metabolites. The cumulative results from 3-4 independent experiments with 5 replicates each is shown.

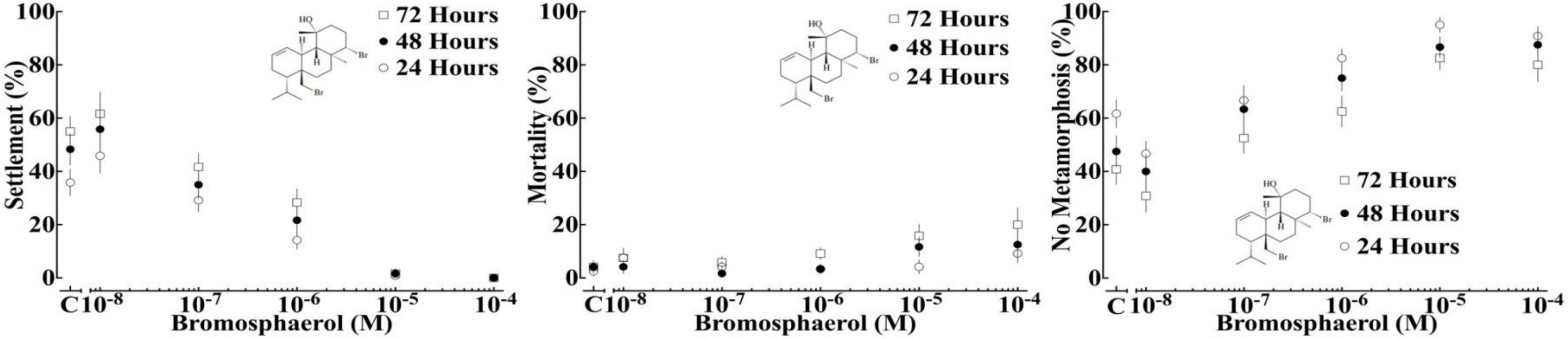

Figure S4. Results of settlement bioassays of *A. amphitrite* cyprids in the presence of varying concentrations of bromosphaerol. Values are expressed as the percentage of animals that settled (settlement) or did not react to light mechanical stimuli (mortality) or neither settled nor were dead (no metamorphosis) from the total number of animals placed in a well of a 24-well tissue culture plate with varying concentrations of bromosphaerol. The cumulative results from 3 independent experiments with 5 replicates each is shown.

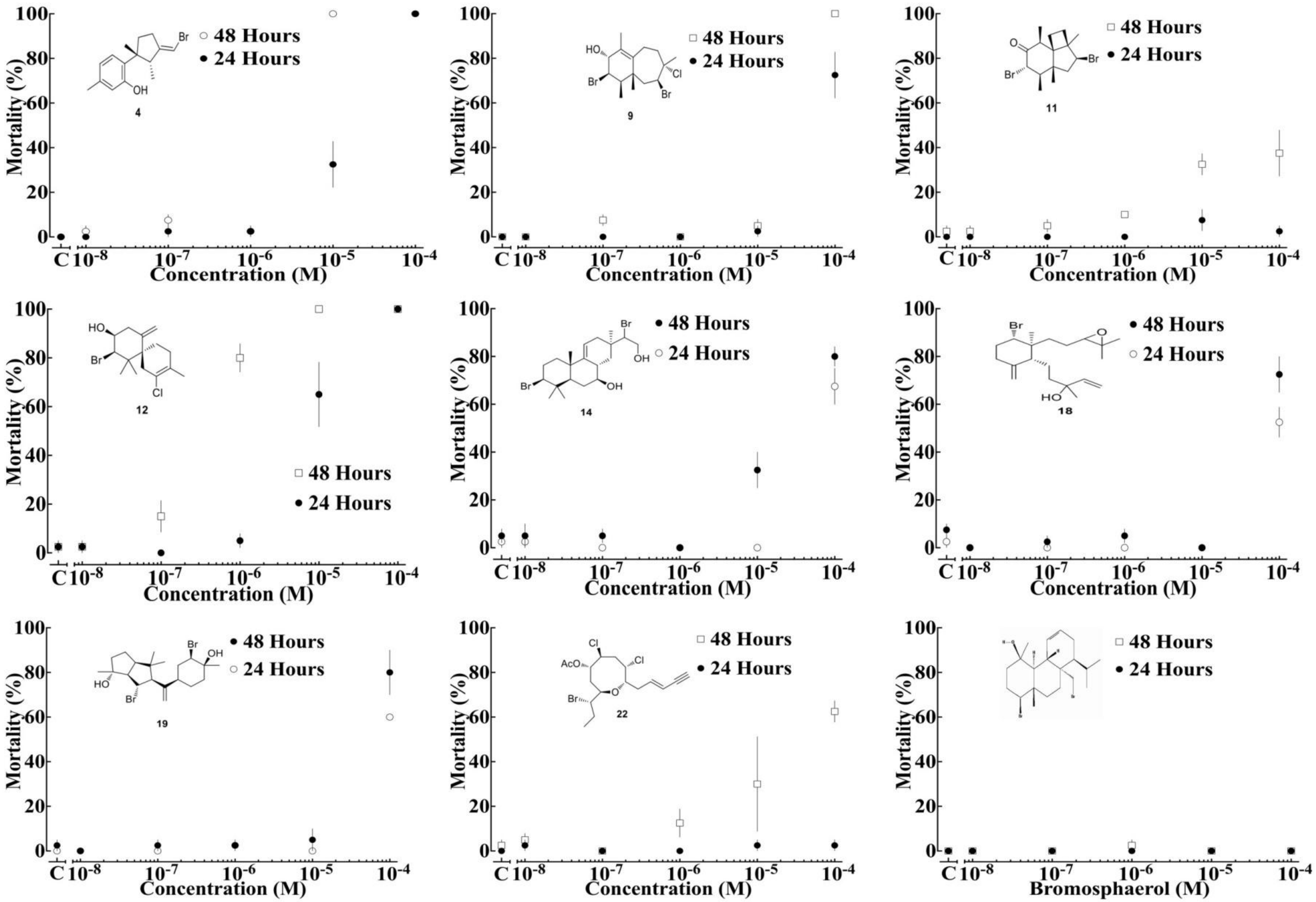

Figure S5. Results of *Artemia salina* toxicity assays in the presence of 8 secondary metabolites isolated from *Laurencia* and *Aplysia* species and bromosphaerol. Values are expressed as the percentage of animals that showed no movement of the appendages from the total number of animals placed in a well of a 24-well tissue culture plate with varying concentrations of the indicated metabolites. The cumulative results from 3 independent experiments with 5 replicates each is shown.

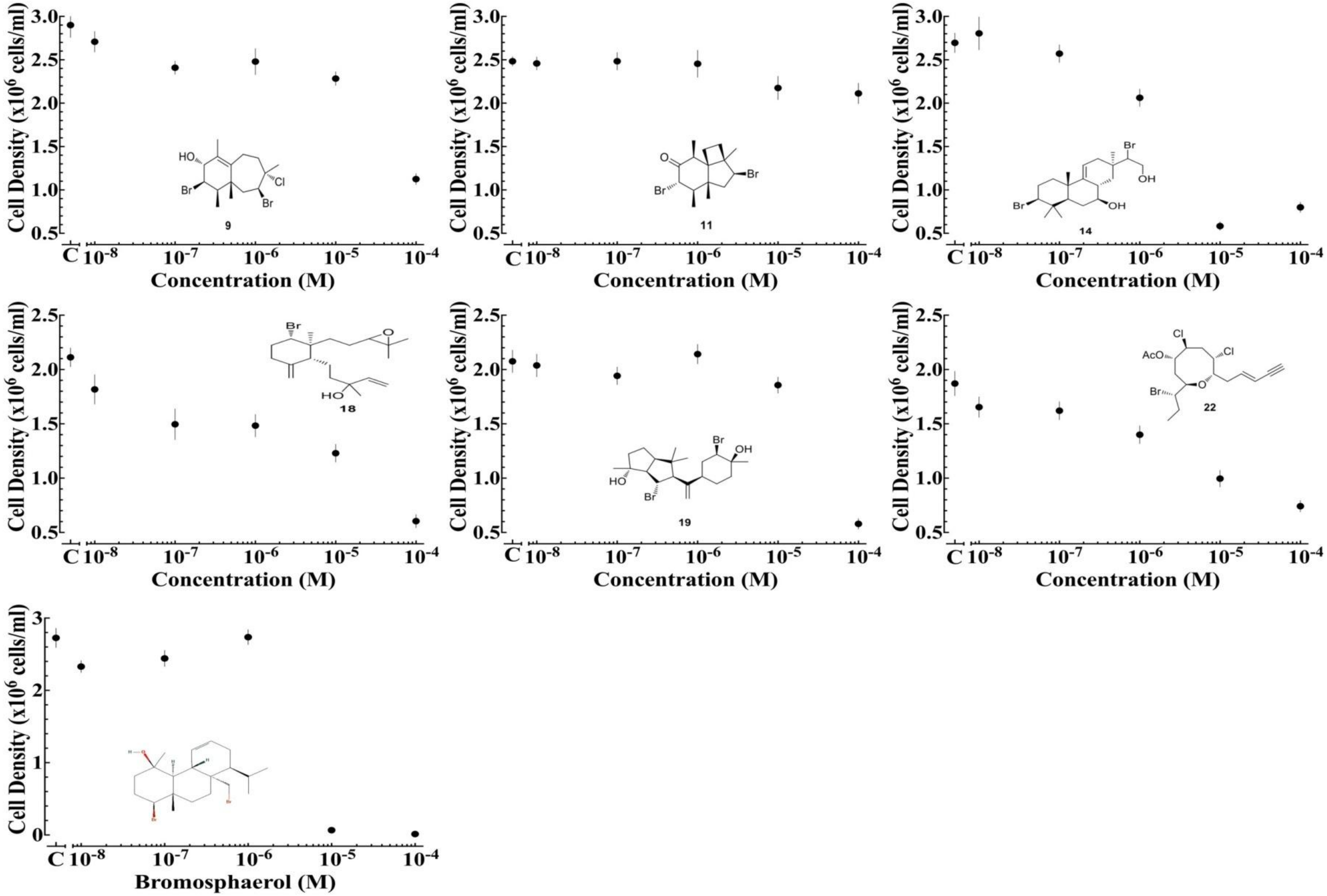

Figure S6. Results of *Chaetoceros gracilis* toxicity assays in the presence of 6 secondary metabolites isolated from *Laurencia* and *Aplysia* species and bromosphaerol.

Values are expressed as cell density of *Chaetoceros gracilis* diatoms in the presence of varying concentrations of the indicated metabolites for 96 h. The cumulative results from 3 independent experiments with 5 replicates each is shown.

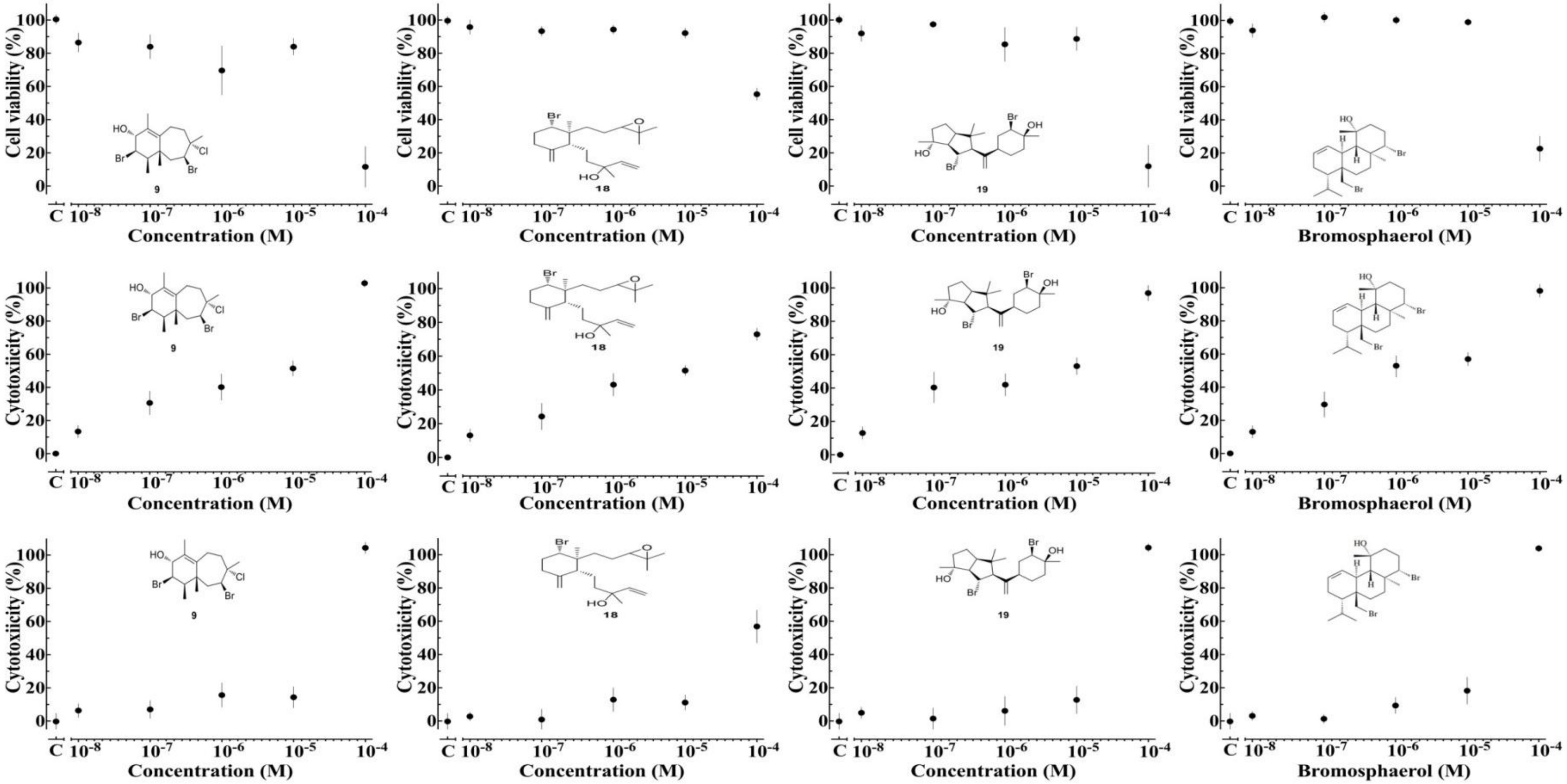

Figure S7. Results of RTL-W1 cell viability assays in the presence of 3 secondary metabolites isolated from *Laurencia* and *Aplysia* species and bromosphaerol. Values are expressed as % cell viability or cytotoxicity (see Materials and Methods for details) in the presence of varying concentrations of the indicated metabolites. The cumulative results from 3 independent experiments with 5 replicates each is shown.

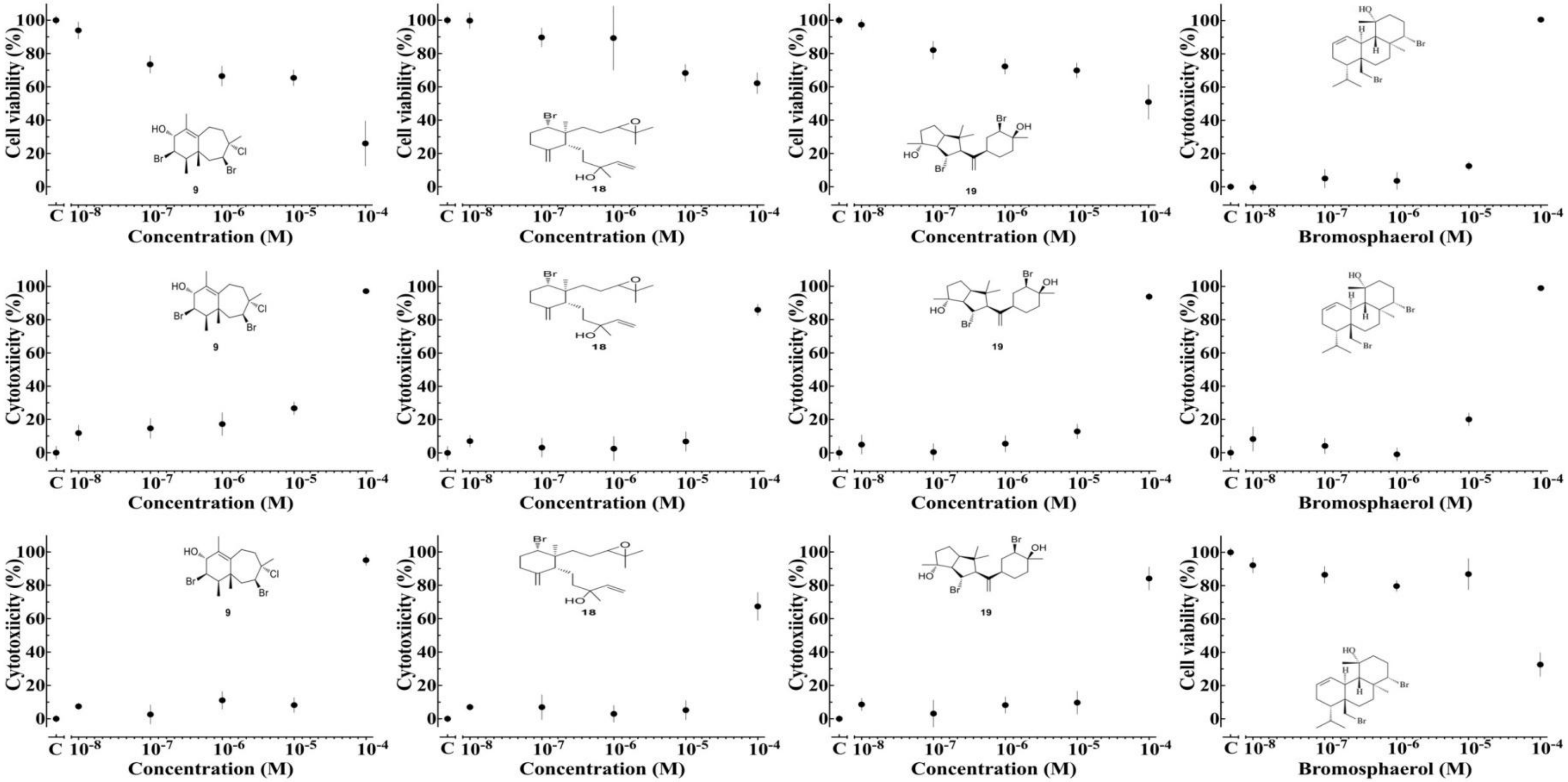

Figure S8. Results of HEK293 cell viability assays in the presence of 3 secondary metabolites isolated from *Laurencia* and *Aplysia* species and bromosphaerol. Values are expressed as % cell viability or cytotoxicity (see Materials and Methods for details) in the presence of varying concentrations of the indicated metabolites. The cumulative results from 3 independent experiments with 5 replicates each is shown.

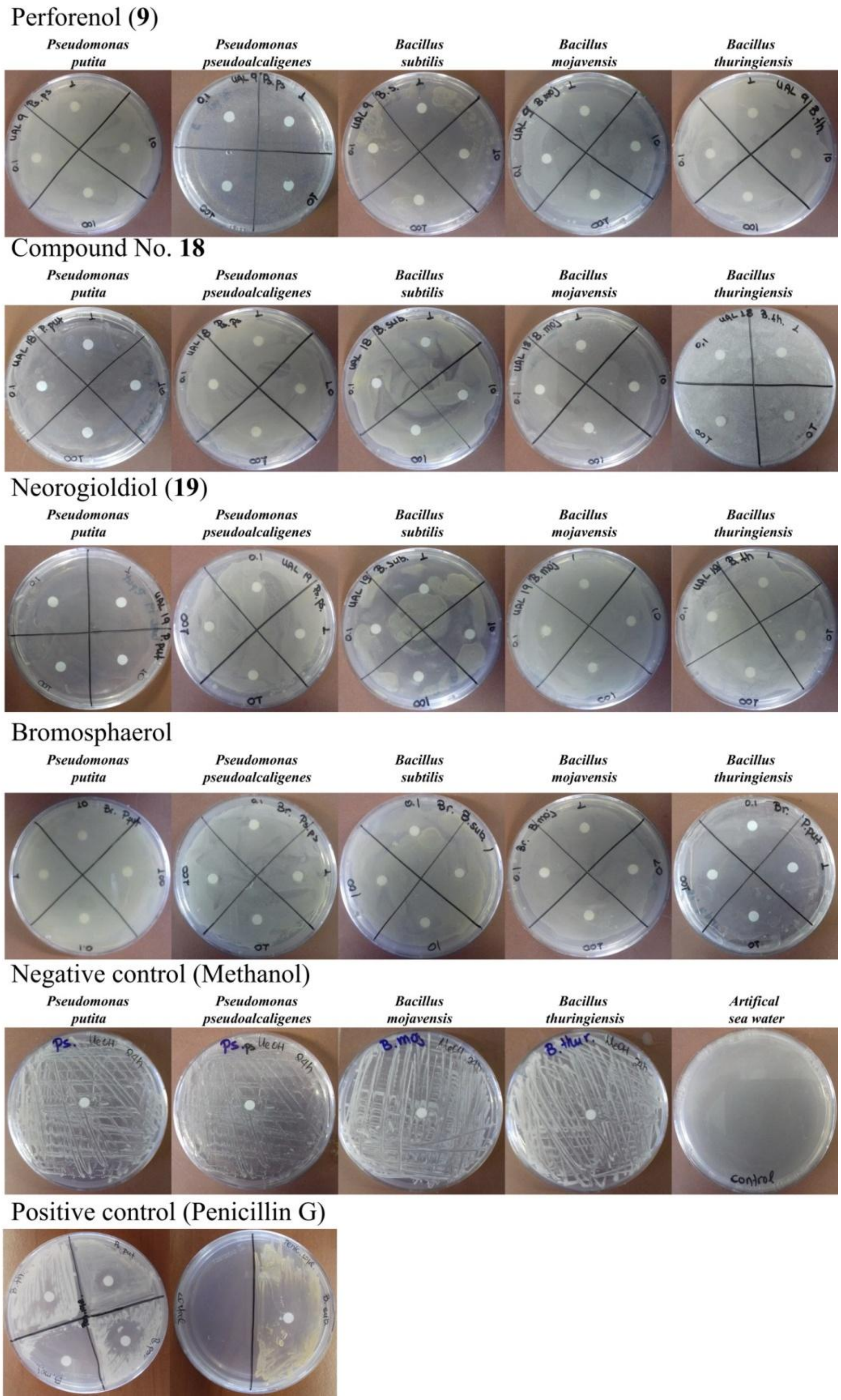

Figure S9. Results of marine bacteria growth assays in the presence of 3 secondary metabolites isolated from *Laurencia* and *Aplysia* species and bromosphaerol.

Marine agar plates (Zobel marine agar medium) were split in 4 sections (each corresponding to a serial dilutions (0.1, 1, 10, 100 μM) of each tested metabolite) and a sterile filter paper disc (4 mm) (GF/F; Whatman), loaded with a 40 μL sample of each tested metabolite, was placed on each section of the agar plates that were then seeded with a single strain of bacteria and incubated for 24 h in 25°C (see Materials and Methods for details). Indicative plates from 3 independent experiments with 5 replicates each are shown.
